# Honor Conferred

**Published:** 1861-11

**Authors:** 


HONOR CONFERRED.—His Majesty the King of Denmark has been pleased to confer the honor of knighthood of the Order of the Danneberg upon JAMES SYME, ESQ., Professor of Clinical Surgery in the University of Edinburgh.

